# Defining neighborhood boundaries in studies of spatial dependence in child behavior problems

**DOI:** 10.1186/1476-072X-12-24

**Published:** 2013-05-03

**Authors:** Margaret O’Brien Caughy, Tammy Leonard, Kurt Beron, James Murdoch

**Affiliations:** 1 University of Texas School of Public Health, Dallas Regional Campus, 5323 Harry Hines Blvd, BL10.204, Dallas, TX, 75390-9655, USA; 2 University of Texas at Dallas, Department of Economics, School of Economics, Political & Policy Sciences, 800 West Campbell Road, GR31, Richardson, TX, 75080-3021, USA

**Keywords:** Neighborhood, Child behavior problems, Peer relationships

## Abstract

**Background:**

The purpose of this study was to extend the analysis of neighborhood effects on child behavioral outcomes in two ways: (1) by examining the geographic extent of the relationship between child behavior and neighborhood physical conditions independent of standard administrative boundaries such as census tracts or block groups and (2) by examining the relationship and geographic extent of geographic peers’ behavior and individual child behavior.

**Methods:**

The study neighborhood was a low income, ethnic minority neighborhood of approximately 20,000 residents in a large city in the southwestern United States. Observational data were collected for 11,552 parcels and 1,778 face blocks in the neighborhood over a five week period. Data on child behavior problems were collected from the parents of 261 school-age children (81% African American, 14% Latino) living in the neighborhood. Spatial analysis methods were used to examine the spatial dependence of child behavior problems in relation to physical conditions in the neighborhood for areas surrounding the child’s home ranging from a radius of 50 meters to a radius of 1000 meters. Likewise, the spatial dependence of child behavior problems in relation to the behavior problems of neighborhood peers was examined for areas ranging from a radius 255 meters to a radius of 600 meters around the child’s home. Finally, we examined the joint influence of neighborhood physical conditions and geographic peers.

**Results:**

Poor conditions of the physical environment of the neighborhood were related to more behavioral problems, and the geographic extent of the physical environment that mattered was an area with a radius between 400 and 800 meters surrounding the child’s home. In addition, the average level of behavior problems of neighborhood peers within 255 meters of the child’s home was also positively associated with child behavior problems. Furthermore, these effects were independent of one another.

**Conclusions:**

These findings demonstrate that using flexible geographies in the study of neighborhood effects can provide important insights into spatial influences on health outcomes. With regards to child behavioral outcomes, specifically, these findings support the importance of addressing the physical and social environment when planning community-level interventions to reduce child behavior problems.

## Background

In the U.S. Surgeon General’s report on children’s mental health [1], behavior problems in children were identified as being responsible for a significant reduction in quality of life for children between ages 1 and 19 due to increased risk of academic failure and high school drop-out, increased delinquent behavior in adolescence, and reduced productivity in adulthood [1-5]. Furthermore, child behavior problems generate significant costs to society. Knapp (2003 as cited by the [6] reported that children with conduct disorders generated £70,000 (approximately $109,000) each in additional costs between the ages of 10 and 27 primarily related to the criminal justice and education systems. The objective of the present study was to extend the analysis of neighborhood effects to more explicitly examine spatial dependencies in child behavior problems related to neighborhood physical conditions as well as to neighborhood social condition measured by the levels of behavior problems reported for peers in the neighborhood.

Dodge and Pettit (2003) provide a summary of the literature regarding risk factors for chronic child behavior problems. In the family domain, family poverty, parental psychological distress, and parenting behavior characterized by low sensitivity and harsh/inconsistent discipline are associated with higher rates of behavior problems. In the peer domain, exposure to aggressive peers during early childhood and affiliation with delinquent peers during later childhood and adolescence are also associated with trajectories of heightened behavior problems. Most intervention efforts to prevent or treat behavior problems in children and youth have focused on family-level interventions, school-level interventions, or combinations of such approaches [7-13]. Although many have demonstrated significant effects in reducing behavior problems, none have yet demonstrated the ability to translate those intervention effects into population-level reductions in rates of child behavioral problems. Dodge [14] argues that the inability of existing intervention efforts to successfully “scale-up” to population-level initiatives results from a failure to adequately consider the impact of community context in modifying the impact of interventions targeting children, families, and/or schools.

### Neighborhood determinants of child behavior problems

A substantial body of research has documented that the physical and social conditions of the communities in which children live are associated with significant differences in child behavior problems over and above individual differences in child and family characteristics. Neighborhoods characterized by concentrated economic disadvantage, physical disorder, and low social cohesion are associated with higher rates of both externalizing and internalizing problems among children and youth [15-21]. Although much of the research on neighborhood effects on child behavior problems is limited by the lack of randomization of families to neighborhoods of varying conditions, there is evidence from an experimental housing voucher program that adolescent boys randomized to the treatment condition, which allowed them to move to lower poverty neighborhoods, showed significant reductions in anxiety and depression [22]. Furthermore, there is evidence neighborhood conditions not only have a direct effect on child behavior problems but also an indirect effect by moderating the impact of family-level risk factors [23-29]. In some instances, neighborhood factors have been found to play protective roles, buffering children against the impact of family-level risk, and in others, neighborhood characteristics have been found to exacerbate family-level risk factors.

### Neighborhood peer influences on child and youth behavior

One aspect of the neighborhood social environment receiving limited attention in the research literature is that of geographic peers. By “geographic peers,” we refer to other children and youth living in geographic proximity to the target child who may or may not interact socially with the target child. The vast majority of research on peer influences on child behavior problems has focused on the relation of exposure to aggressive and/or delinquent peers in the child’s social group to maladaptive outcomes such as drug use, sexual risk taking, or academic failure [30-32]. In most cases, the child’s social group is defined as the children with whom s/he regularly interacts as identified by the target child. Neighborhood research that has included a focus on peer influences either examined peers as another factor affecting child behavior jointly with neighborhood factors [17,18,33] or focused on affiliation with deviant peers as an outcome of neighborhood influences [34-37]. Although theoretical perspectives have emphasized the importance of community norms as a mechanism of neighborhood influences [38-40], neighborhood research has not incorporated measures of the behavior of geographically proximal peers as a potential neighborhood-level influence.

An important concept when considering the influence of peers in the neighborhood is that of a “social multiplier effect” [41]. If a child’s behavior affects the behavior of the peers proximal to him in the neighborhood, then those effects will be reciprocal. That is, Child A’s behavior will affect Child B’s behavior, who will in turn affect Child A’s behavior, and so on and so forth. In quantifying the effect of Child A’s behavior on Child B, one must distinguish between the direct effect, or the effect of that first transaction, from the total effect, which estimates the effect of all reciprocal transactions. These reciprocal transactions are the “social multipliers” which increase the magnitude of the effect of one individual on another. We study the presence of social multipliers whereby the behavior of geographically close peers is endogenously related. The geographic-based social multiplier has received little attention with regards to child behavior problems and represents an important new avenue for further research aimed at understanding how policy might more cost effectively reduce public expenditures on problems related to child conduct disorders.

### Measurement issues in studying neighborhood effects

Throughout the extant literature on neighborhood effects is an unresolved issue as to how to best operationally define neighborhood boundaries. When addressing the question about how to measure neighborhood characteristics, Guo and Bhat [42] state that “we should measure what matters to people over the area that really matters to people” (p. 31). Most often, however, neighborhood researchers default to administrative boundaries of neighborhoods such as census tracts or census block groups. However, the utilization of census tracts and census block groups represents a definition of convenience rather than one based on theoretical considerations of how neighborhoods affect individual health and well-being. Accurately identifying the neighborhood of influence for a particular outcome is critical for the formulation of effective public policy targeting neighborhood conditions. To date, however, few investigators studying the effects of neighborhood context on individual health and well-being have utilized more sophisticated spatial definitions of neighborhoods that go beyond the simple utilization of census tract or census block group boundaries, and the examples which we do find rarely test the implications of alternative neighborhood specifications.

Chaix and colleagues [43] used hierarchical geostatistical models to analyze spatial variations in drug use in Malmö, Sweden independent of administrative boundaries. They compared this analysis with a more traditional, multilevel approach and found associations were stronger in the spatial analytic approach. In contrast, Ross and colleagues [44] did not find using “natural neighborhoods” provided different results compared with using more traditional, census-defined neighborhoods in studying neighborhood effects on health in Montreal. The most developed utilization of spatial analysis in health research has been in the area of physical activity in which neighborhood boundaries are often defined by a prescribed distance from the individuals home [45,46] and spatial sampling of the built environment has been given more attention [47]. However, we still find relatively few examples in which multiple neighborhood definitions are compared and contrasted in order to statistically determine the extent of the relevant neighborhood boundary for the research question at hand. One example moving in this direction is the work of Moudon and colleagues [48] who used survey reports of physical activity and linked it to objective neighborhood characteristics to determine the threshold for distances between residences and neighborhood physical features that supported walking. In what follows, we test multiple definitions to determine the scope over which neighborhood effects manner.

### Limitations of neighborhood research to inform policy and prevention

If neighborhood physical or social conditions are related to children’s behavior problems, then policy and interventions should address this mode of influence. A better understanding of how neighborhood social and physical conditions directly contribute to child behavior problems would enhance our ability to scale-up evidence-based interventions. For example, the assessment of community characteristics could be incorporated into evaluations of family-level and school-level intervention programs to examine how these characteristics may moderate program impacts. Such information would better inform dissemination efforts of effective programs to new communities.

In addition, neighborhood-level outcomes could be examined as a potential consequence of individually-focused intervention programs. Dodge (2011) identifies low rates of intervention uptake as one of the factors limiting the population-level impact of family- and school-based interventions to reduce child behavior problems. However, it is possible the population-level impact of an intervention could be propagated via peer networks. Peers have been identified as an important source of influence in child behavior problems [17,26], and the propagation of behaviors by peers has been suggested as one of the mechanisms of neighborhood effects on youth behavior [49]. Limiting the evaluation of interventions to measurements of outcomes in those directly touched by the intervention may underestimate the population-level impact of the intervention unless one also examines how change in an individual child may result in change for other children in the neighborhood not directly touched by the intervention via the social multiplier effects described above. Although economic analyses of early intervention programs to improve child behavioral and academic outcomes indicate long-term benefits exceed costs [50-52], these analyses may be underestimating the benefits of such programs if impacts on peers in the neighborhood are not taken into account. Additionally, if children in the treatment and control groups are members of overlapping peer networks, treatment effects measured by comparing differences across the two groups may be diminished because social multipliers are producing “treatment externalities” in the control group.

The objective of the present study was to extend the analysis of neighborhood effects to more explicitly examine spatial dependencies in child behavior problems related to neighborhood physical conditions as well as to the levels of behavior problems reported for peers in the neighborhood. This study takes advantage of a unique data set containing measures of neighborhood conditions in which neighborhood physical conditions were documented at the smallest possible geographic area, the parcel. The high level of resolution at which neighborhood data were collected affords us with the ability to examine the spatial dependence of parent-reported child behavior problems as related to neighborhood physical conditions independent of standard administrative boundaries such as census tracts or block groups. In addition, we examine the spatial dependence of child behavior problems associated with the behavior of peers in the neighborhood and examine whether it is independent of the spatial effects of neighborhood physical conditions.

## Methods

Data for this study were drawn from a larger study examining the effects of publicly driven investment on social, economic and health indicators in a low-income (median household income = $30,512), primarily African American (70%) neighborhood in a large city in the Southwest. Participants were recruited during a door-to-door survey of a stratified multi-stage random sample of households in the community as well as additional sampling of other neighborhood households with an address matching that of a child registered in the local public school district. Neighborhood families with at least one child aged 5–13 were preferentially selected into the study. Participating families came to a centralized center in the community and completed a series of survey questions for each school-age child in their household. A total of 141 participants reported data for a total of 261 children. Just under half of these 141 participants (63, 44.7%) reported on only one child in their household; 47 (33.3%) reported on two children, 20 (14.2%) reported on three children, and 11 (7.8%) participants reported on four children. The characteristics of the 261 children are displayed in Table 1. Most of the children in the sample were elementary school-age, with just under half (48.7%) between the ages of 6 and 9 years. The sample included 19 children which were in grades 7–12; the results reported are robust to the inclusion/exclusion of the older children in the sample. The majority of the sample (80.7%) was African American, non-Latino, and 14.5% of the sample was Latino. Consistent with the study community, children in the sample were primarily from low income families. Almost half (46.2%) were living in families with a reported annual income of less than $10,000.

**Table 1:** Sample characteristics (N = 261)

	**N**	**%**
Age		
Early childhood (4–5)	54	20.7
Middle childhood (6–9)	127	48.7
Pre-teen (10–12)	53	20.3
Early teen (13–15)	20	7.7
Late teen (16–18)	7	2.7
Gender		
Boy	117	44.8
Girl	130	49.8
Not reported	14	5.4
Child race/ethnicity		
African American, non-Latino	213	81.6
Latino	36	13.8
European American, non-Latino	2	.8
American Indian, non-Latino	1	.4
Not reported	9	3.4
Parent education		
Less than high school	79	30.3
High school/GED	94	36.0
More than high school	66	25.3
Not reported	22	8.4
Family income (per year)		
Less than $10,000	119	45.6
$10,000-$30,000	94	36.0
$30,000-$50,000	27	10.3
More than $50,000	16	6.1
Not reported	5	1.9

### Measures

Parents reported on child behavioral competence using the Behavior Problems Index (BPI) [53]. For children under the age of 12, the BPI is comprised of 27 items, and for children age 12 years and older, the BPI is comprised of 25 items. For both age groups, the caregiver responds to each item as “Often true,” “Sometimes true,” or “Not true” for the target child. In this sample, the internal reliability of the BPI was .94 for children under the age of 12 and .93 for children age 12 years and older. Each item is dichotomized (Often/Sometimes versus Not True), and a total BPI score was computed by averaging the items and then standardizing. Higher BPI scores indicate more behavior problems.

Data on neighborhood physical conditions were collected using an instrument adapted from the Neighborhood Observation Checklist [54]. Described more fully elsewhere (author reference), observations were conducted for each face block and parcel in the study neighborhood. The study neighborhood had an area of approximately 2000 acres, had a population of approximately 20,000, and was comprised of seven census tracts and thirty-two block groups. In a residential neighborhood, parcels are the piece of property that includes the house, surrounding yard, and all of the property that the homeowner owns and is assessed for tax purposes. As such, the parcel represents the smallest, non-reducible geographic unit of the neighborhood. A list of the items assessed as part of the observation is included in the Additional file 1. Some items were recorded at the parcel level, while others were recorded for the face block, which consisted of other parcels on either side of the street between two intersections. Parcel usage codes were assigned to all parcels in the neighborhood, while other parcel condition codes were only collected for single-family residence parcels. Observations were directly entered into a geocoded data base using laptop and tablet computers running *ArcPad* software (ESRI, http://www.esri.com). A total of 11,552 parcels and 1,778 face blocks were observed over a period of five weeks. Inter-rater reliability of the observations was assessed using 10% of observations that were rated by a second pair of raters; average inter-rater reliability was .91 (range: .66 to .998). Only the parcel-level data will be used in this analysis.

A composite index of the physical condition of each single family residence parcel, derived in (author reference), was used comprised of housing condition, peeling paint, boarded windows, unkempt lawn, fence in poor shape, and trash in yard. Each item was coded 1 to indicate deleterious conditions for that item. An exploratory factor analysis of the six items identified only one factor with an eigenvalue over 1 (3.54), and a confirmatory factor model indicated that a one-factor model was a reasonable fit for the data, χ^2^ (3) = 7.66, *p* = .054, CFI = 1.00, root mean square error of approximation (RMSEA) = .012. The six items were summed to create the composite index that we will henceforth refer to as *parcel condition*. The average value for *parcel condition* was 1.01 (SD = 1.14; range 0 to 6). A total of 2792 parcels (42.9%) had no negative physical conditions observed, whereas 1880 (28.9%) had 1 item, 1131 (17.4%) had 2 items, and 710 (10.9%) had 3 or more.

The parcel condition data was used to create composite neighborhood physical condition indicators for geographies of differing sizes. For each parcel where a child in our study lived, we calculated the average of *parcel condition* for circles of varying radii: 50 meters, 100 meters, 200 meters, 400 meters, 600 meters, 800 meters and 1000 meters

Other covariates collected as part of the household survey with participants included child gender, child race/ethnicity, number of children 18 and under in the household, parental education, household income, length of residence in the neighborhood, and parental health status. In the statistical analysis, we control for child race/ethnicity (Hispanic vs. non-Hispanic black). Parental education was included as a binary variable indicating parents with less than a high school education; and household income is controlled for with three binary variables indicating income categories of $10,000 to $19,999, $20,000 to $29,999, and income greater than or equal to $30,000. The omitted category was household income less than $10,000. Parental health status was based on the response of the adult household member who completed the survey questionnaire to the question “In general your health is …excellent, very good, good, fair, or poor.” The *good health* binary variable used in the model indicated responses of excellent, very good or good.

### Ethics considerations

All data collection procedures and measures for the study were reviewed and approved by the Institutional Review Board of the University of Texas at Dallas (*approval # 08–33, dates: 20 June 2008 – 30 July 2013*).

### Statistical analysis

To examine the role of neighborhood physical and social (neighborhood peers) environment on standardized child BPI, we characterize these effects as endogenous, exogenous or correlated effects [41]. These terms have become standard in the economics literature examining neighborhood and peer effects and each has a very specific meaning related to the mechanism that generates the association. Correlated effects occur when individual behavior is similar because of shared *individual* characteristics that are correlated with behavior. An example of a correlated effect in our study might be the negative association between income and child behavior problems [55-58]. Income would be classified as producing a correlated effect because children with low-income often live in the same neighborhoods; in other words the correlation between income and child behavior produces similar child behavior within a neighborhood. Our model included an extensive set of socio-demographic controls to account for these potential correlated effects in child BPI. The second type of effect we examine is exogenous. Exogenous effects are defined as *group or neighborhood* measures related to the outcome variable that are exogenous to the outcome measure itself. They exist because individuals are exposed to similar environments or other factors. For example, child behavior may be influenced by poor neighborhood upkeep and neighborhood upkeep and child behavior are exogenously determined. Our unique measure of neighborhood condition allows us to examine the exogenous effects of the local neighborhood, and it allows us to test the geographic extent of the neighborhood influence. Finally, endogenous neighborhood effects are defined as *group or neighborhood* measures related to the outcome variable that are endogenously determined with the outcome variable. They occur because individuals are influenced by their peers, or reference group. We used the behavior of other children in our study who live in close proximity as the “geographic peers,” and we tested if endogenous effects are present within the geographic peer network. Independent identification of endogenous, exogenous and correlated effects has been given much attention in the econometrics literature [59,60]. In what follows, we incorporate these methods to examine each of the independent effects.

The statistical analysis proceeded in three phases. First, we examined the relation between child BPI and physical neighborhood condition (exogenous effects) while controlling for individual socio-demographic characteristics (correlated effects). We estimated seven models with child BPI as the dependent variable, the full set of socio-demographic controls, *X*, and a single measure of neighborhood physical condition, *N:*

(1) $$\text{BP}I_{i}=\beta x_{i}+\gamma N_{i}+\in_{i}$$

Each of the seven models used a different geographic scale for the neighborhood physical condition variable, *N*. The geographic scale was based on circles of increasing radius for which the average *parcel condition* was calculated: 50 meters, 100 meters, 200 meters, 400 meters, 600 meters, 800 meters and 1000 meters. The geographic scope of neighborhood physical condition that affected child BPI was examined by comparing the coefficient estimates of *γ* in the seven models. Each of the measures in (1) is observed at the individual level, indicated by the subscript *i* in (1). Despite the fact that individuals living in close proximity may have highly overlapping measures of neighborhood condition, the variable is unique for each individual because neighborhood condition (*N*) is measured at a given radius around each individual’s home. This captures the fact that two children living on the same block (for instance) might both live near a “bad” part of the neighborhood, but the child living closer to the “bad” area will have a larger portion of their neighborhood buffer zone within the “bad” area and hence a worse measure of neighborhood condition.

The next phase of the statistical analysis examined the relation between child BPI and the BPI of the child’s geographic peers (the endogenous effect). The key independent variable in these models was the average BPI of the other children in the neighborhood defined according to one of the geographic scales as before (i.e. a circle of radius 50 meters, 100 meters, 200 meters, 400 meters, 600 meters, or 800 meters):

(2) $$\text{BP}I_{i}=\rho W_{i}\text{BP}I_{i}+\beta x_{i}+\in_{i}$$

Once again, the subscript *i* indicates that each measure is observed for each individual. Here, *W*_*i*_ is the spatial weights matrix and identifies neighbors of each child, excluding the child herself. We identified all neighbors within the prescribed neighborhood geography for each child, and the corresponding element of the spatial weights matrix was coded 1; all other elements of *W* were 0. The weights matrix was then standardized so that each row summed to 1, and so that the product of *W* and *BPI* returns a vector of average neighborhood BPI. The model described in (2) is a classic spatial lag model and implies non-linear relationships between the dependent variable, *BPI*, and the independent variables. Unbiased, efficient estimates for these types of models may be obtained using maximum likelihood estimation [61,62]. The primary goal of this analysis was to test for a positive statistically significant estimate for *ρ*, the geographic peer effect.

The final analysis phase investigated the contemporaneous impact of physical neighborhood condition along with the BPI of geographic peers:

(3) BPI=ρWBPI+βx+γN+∈

Here we simultaneously control for correlated (*X*), exogenous (*N*) and endogenous (W*BPI) effects. In (3) we wish to understand the degree to which the estimated values of *γ* and *ρ* change from those estimated in (1) and (2), respectively, when both neighborhood physical condition and the BPI of geographic peers are considered together.

## Results

In all of the multivariate models reported, we find that child gender and household income have a statistically significant relationship with child BPI. Females and children in households with income greater than or equal to $30,000 annually tend to have fewer behavioral problems. In what follows, we focus our attention on the independent variables of greatest interest: neighborhood physical condition and the average BPI of geographic peers. After the basic analyses are reported, we describe a series of sensitivity analyses.

### Analysis of the impact of neighborhood physical condition on child behavior problems

We estimated equation (1) with ordinary least squares varying the condition index from 50 meters to 1000 meters while including in each model all previously described covariates. In all OLS regressions, robust standard errors are reported that also were adjusted for clustering of children within families. Neighborhood condition in the 400-, 600-, and 800- meter circles were statistically significant.

We tested the three significant circle sizes using a Wald test to determine if their estimated magnitudes were the same. We found the null of equality across the three parameter estimates could not be rejected, *χ*^2^ (2) = 2.73, *p* = .26. Similarly, we tested the other two significant variables across the three regressions with the significant circle sizes and found no statistical evidence that female, *χ*^2^ (2) = 3.40, *p* = .18, or income greater than $30 thousand, *χ*^2^ (2) = 1.81, *p* = .41, were different at the three geographies. As a robustness check, we also tested whether the parameter estimate of the (non-significant) circle size at 200 meters was the same as that of the 400 meter circle size and rejected the null hypothesis of equality, *χ*^2^ (1) = 4.83, *p* = .03. We also tested 1000 meters which, while having a non-significant parameter estimate, still was indistinguishable statistically in value to smaller sizes down to 400 meters.

Thus, with evidence that the 400 meter and larger circles were statistically equivalent, we report the 400 meter model results in Table 2. Our findings, then, based on OLS, were that a one standard deviation increase in negative physical conditions in the neighborhood was expected to lead to about a one-quarter standard deviation increase in the BPI at the 400-meter and above circle geography.

**Table 2:** OLS results for 400-meter radius circle size (n = 208)

**Variable**	**b**	**se(b)**	**t**
Gender (1 = girl)	-.363	.138	−2.64**
Ethnicity (1 = Latino)	.212	.295	-.72
Parental education (1 = <HS)	.103	.217	-.47
Family income $10-20 k	-.187	.208	-.90
Family income $20-30 k	.162	.357	-.45
Family income $30 k+	-.423	.182	−2.33*
No. years in neighborhood	.001	.005	-.26
Total children in household	-.042	.048	-.87
Parental health status (1 = good/excellent)	-.023	.190	-.12
Child age	.038	.023	−1.64
Condition (1 = poor)	.279	.106	−2.63**
Constant	.054	.359	-.15

*p < .05; **p < .01.

### Analysis of the impact of geographic neighborhood peers on child behavior problems

The first step in analyzing the impact of geographic neighborhood peers was specifying a weight matrix that determined the relevant geographic peer group. As before, we tested multiple geometries for the spatial weights matrix to correspond with neighborhood definitions at varying distances from the child’s home. Because the spatial weights matrix must be specified such that every observation in the sample has at least one neighbor in the sample, the smallest neighborhood geometry that we could specify for the spatial weights matrix was a circle with a 255-meter radius. For larger geometries, we used the same conventions as before (400 m, 600 m and 800 m).

We first tested for spatial correlation in the errors of the previous ordinary least-squares regression models by calculating Moran’s *I* statistics [61]. The Moran’s *I* statistic is a measure of spatial correlation. A statistically significant Moran’s I in our study indicates that the residuals of nearer observations are more likely to be similar than observations located further apart. As condition at 400 m, 600 m and 800 m were found to be statistically significant and have similar associations with child BPI, we focused on these models. In this application, the spatial weights matrix defines the geographic scope over which the spatial correlation is hypothesized to occur. Table 3 presents the Moran’s *I* statistic, and the associated *p*-value for each of the different combinations of neighborhood condition geography and weight matrix geography. Two interesting results emerged. First, when we did not control for neighborhood physical condition, the Moran’s *I* statistic was statistically significant for each of the different weight matrix geographies. However, when we did control for the neighborhood physical condition, we found that only the weight matrix based on a 255 meter radius resulted in a statistically significant Moran’s *I*. Thus, for spatial weights matrices based on larger neighborhood definitions, we failed to reject the null hypothesis that there was no spatial correlation remaining in the model’s residuals once we controlled for the neighborhood physical condition. This suggests the influence of geographic peers is present after accounting for the exogenous neighborhood effect only for very near geographic peers.

**Table 3:** **Moran's*I*statistic for different definitions of neighborhood geography**

	**Weight matrix geography**			
**Neighborhood condition geography**	**255 m**	**400 m**	**600 m**	**800 m**
No control for neighborhood condition	.167***	.077+	.045*	.042**
400 m	.072+	.002	-.020	-.003
600 m	.108**	.033	.008	.009
800 m	.124**	.043+	.018	.013

+p < .10; *p < .05; **p < .01; ***p < .001.

Next we estimated (2) and (3) with maximum likelihood algorithms [62] for each of the neighborhood definitions for which the Moran’s *I* test rejected the hypothesis of no spatial correlation. For (2) we did not control for neighborhood physical condition, and Moran’s *I* tests were statistically significant at all neighborhood geographies. The results for these models are presented in Table 4.

**Table 4:** Coefficient estimates for spatial autoregressive models without controls for neighborhood condition

	**Weight matrix geometry**											
	**255 m**			**400 m**			**600 m**			**800 m**		
	**b**	**se(b)**	**t**	**b**	**se(b)**	**t**	**b**	**se(b)**	**t**	**b**	**se(b)**	**t**
Constant	.056	.311	.18	.045	.321	.14	.046	.307	.15	.067	.305	.22
Gender (1 = girl)	-.419	.129	−3.24^**^	-.420	.131	−3.20^**^	-.408	.131	−3.11^**^	-.389	.131	−2.97^**^
Ethnicity (1 = Latino)	.344	.190	1.81^+^	.353	.193	1.83^+^	.334	.193	1.73^+^	.341	.193	1.77^+^
Parental education (1 = <HS)	.146	.140	1.04	.118	.142	.83	.150	.142	1.06	.136	.142	.96
Family income $10-20 k	-.235	.163	−1.44	-.244	.165	−1.48	-.225	.165	−1.36	-.227	.164	−1.38
Family income $20-30 k	-.001	.303	.00	.058	.232	.25	.054	.225	.24	.070	.226	.31
Family income $30 k+	-.571	.194	−2.94^**^	-.552	.196	−2.81^**^	-.517	.197	−2.63^**^	-.517	.195	−2.65^**^
No. years in neighborhood	.002	.005	.38	.002	.005	.39	.002	.005	.43	.001	.003	.31
Total children in household	-.047	.035	−1.36	-.051	.035	−1.45	-.052	.035	−1.49	-.057	.035	−1.65^+^
Parental health status (1 = good/excellent)	.058	.149	.39	.051	.150	.34	.045	.150	.30	.053	.151	.35
Child age	.038	.021	1.80^+^	.040	.021	1.88^+^	.039	.022	1.81^+^	.039	.021	1.85^+^
Geographic peer effect (rho)	.229	.080	2.88^**^	.217	.112	1.93^+^	.279	.140	1.99^*^	.374	.158	2.36^*^

+p < .10; *p < .05; **p < .01.

The estimation results presented in Table 5 are based on (3). Here, we analyzed average neighborhood BPI for the 255 m neighborhood only as indicated by the Moran’s *I* tests. Model 1 included the neighborhood physical condition using the 400 m radius and spatial peers defined over a 255 meter radius; Model 2 examined the neighborhood physical condition using the 600 m radius and spatial peers at a 255 meter radius; and finally Model 3 examined a 800 m radius for neighborhood physical condition with spatial peers at a 255 meter radius. When examining the exogenous (neighborhood physical condition) and endogenous (neighborhood peers) effects in the model, we found that in all cases, neighborhood physical condition was statistically significant. The coefficient estimates for geographic peers are positive in each model and statistically significant in Models 2 and 3. Interestingly, as the coefficient estimate for the geographic peers increased in magnitude and statistical significance, the coefficient estimated for the neighborhood condition variable decreased in magnitude and statistical significance. There is likely some trade-off occurring as we vary the geographic scope over which we measured the neighborhood characteristics. Likelihood ratio tests were performed to test the joint significance of controlling for the relationship between neighborhood physical and social characteristics (both the neighborhood condition and the effects of neighborhood peers) for each of the models estimated in Table 5. In all cases the likelihood ratio statistics were statistically significant. The statistical tests indicate that both the neighborhood physical condition and the average behavior problems of geographically close peers are statistically important correlates of child BPI.

**Table 5:** Coefficient estimates for spatial autoregressive models with all covariates

	**Model 1**			**Model 2**			**Model 3**		
	**b**	**se(b)**	**t**	**b**	**se(b)**	**t**	**b**	**se(b)**	**t**
Constant	.071	.309	.23	.065	.310	.21	.060	.207	.29
Gender (1 = girl)	-.360	.129	−2.80^**^	-.377	.129	−2.92^**^	-.395	-.129	−3.06^**^
Ethnicity (1 = Latino)	.217	.190	1.14	.252	.191	1.32	.270	.113	2.40^+^
Parental education (1 = <HS)	.084	.140	.60	.103	.141	.73	.114	.141	.81
Family income $10-20 k	-.168	.162	−1.04	-.201	.162	−1.24	-.220	.162	−1.36
Family income $20-30 k	.130	.224	.58	.074	.224	.33	.041	.228	.18
Family income $30 k+	-.432	.195	−2.22^*^	-.456	.197	−2.32^*^	-.485	.198	−2.45^*^
No. years in neighborhood	.002	.005	.40	.001	.005	.22	.001	.003	.31
Total children in household	-.044	.034	−1.29	-.040	.034	−1.16	-.042	.034	−1.22
Parental health status (1 = good/excellent)	-.008	.160	-.05	.019	.146	.13	.037	.148	.25
Child age	.035	.021	1.70^+^	.035	.021	1.68+	.036	.021	1.73^+^
Average parcel condition (400 m)	.236	.074	3.19^**^	---		---	---		---
Average parcel condition (600 m)	---		---	.183	.073	2.51*	---		---
Average parcel condition (800 m)	---		---	---		---	.137	.072	1.91+
Geographic peer effect (rho)	.110	.088	1.25	.149	.086	1.73+	.179	.084	2.13*

+p < .10; *p < .05; **p < .01.

### Sensitivity analyses

We conducted several analyses to examine the robustness of the findings to alternate model specifications. First, we examined whether there were differences between boys and girls and the relation between conditions and the behavior problem index (BPI) across all geographies. The gender interaction term was non-significant at every circle size. A more rigorous set of constraints was imposed to see whether there was evidence that separate models for girls and for boys were warranted, with the result being that pooling across sex could not be rejected at any circle size. Therefore, all multivariate models are pooled across child gender.

In addition, because the primary dependent variable in the model is standardized child BPI, about 14% of the observed BPI values are zero. As a robustness check, we accounted for the 14 percent pileup at our lowest BPI value by re-estimating our final models using Tobit. The estimated coefficients on our condition indices as well as covariates were almost identical.

Finally, there is also concern that neighborhood socio-economic status may be an important correlate with child BPI that is also correlated with both neighborhood condition and average neighborhood BPI. To examine this potential source of endogeneity, we estimated spatial Durbin models [63] that directly control for average neighborhood income, race, parental education, and health status. The results from these models were very similar to the estimation results reported in Tables 4 and 5 and are available from the authors upon request.

## Discussion

We examine the contemporaneous correlated, exogenous and endogenous effects of child behavior problems for children living in a low-income, ethnic minority neighborhood. Our results suggest an important role for all three paths of influence. The correlated effects indicate that dis-amenities in the physical environment of the neighborhood in which children reside are related to more behavioral problems, and the geographic extent of the physical environment that matters is an area with radius between 400 and 800 meters surrounding the child’s home. Finally, the endogenous effects—measured as the average level of behavior problems of neighborhood peers within 255 meters of the child’s home—are positively associated with child BPI.

These findings provide important policy insights. The vast majority of interventions to prevent or treat child behavior problems have focused on individual and family correlates [7-9,12]. The results of this study support the importance of considering the community context as suggested by Dodge [14] in that significant levels of negative physical conditions in the area surrounding a child’s home are related to higher levels of child behavior problems. Interventions for children living in distressed neighborhoods would do well to coordinate with community-level environmental improvement efforts and/or include assessment of neighborhood conditions in the evaluation of potential treatment moderation effects. Likewise, our results demonstrate the importance of geographic peer-network effects for child behavior problems in the neighborhood, which is another outcome that should be included in program evaluations. The existence of geographic peer effects indicate that “untreated” children likely benefit from living in close proximity to “treated” children.

Additionally, the analysis uncovered important findings regarding the geographic definition of neighborhood that mattered for each type of effect. The boundaries for the physical environment that mattered for child behavior encompassed a much broader area than those for the neighborhood peer (e.g., the social environment). This suggests that policies aimed at improving the neighborhood physical environment should target a larger geography. On the other hand, it also means that improvements to a highly concentrated area of neighborhood environment features that need rehabilitation might provide benefits to residents at a fairly large distance.

The geographic scope over which we found evidence of the endogenous impact of neighborhood peers was much smaller (255 meters). Using the estimates from Model 3, a decrease in the average BPI score of geographic peers within this area by one standard deviation is associated with a reduction in child BPI of .18. Although this effect size would be considered modest [64], it is consistent with the effect sizes for the relation of sensitive parenting with child behavioral competence reported by others [65]. However, this effect size does not account for the social multiplier effect, or the reciprocal influence between a child and his geographic peers. The total effect of a reduction of the average BPI of a child’s geographic peers can be calculated as (1-ρ)^-1^ times the direct effect [66] or 1.23. Thus, the total effect size for a one standard deviation change in average behavior problems of geographic peers is 1.23 for all peers within a 255 meter radius. This is significantly larger than effect sizes reported for behavior problem interventions, which range between .30 and .89 [8,12].

Evidence of the simultaneous endogenous and exogenous effects is highly important for policy analysis and implementation, but the results here should be considered within the limitations of our study. First, the sample is from a single neighborhood, so although our results are supported by numerous studies examining either the neighborhood physical environment [15,19,20,67] or social peers in isolation [68-70] and the development of child behavior problems, the external validity of our contemporaneous estimation of the two types of effects is unknown. Additionally, due to data limitations, our enumeration of the geographic peer network is incomplete. There are many more children in the neighborhood that are not enrolled in our study, and the school peer group of children (for which we do not have complete data) is likely another highly important reference group. We hypothesize inclusion of a more complete peer network would improve the strength of the endogenous effects, but this hypothesis is not testable with our current data. Further, a more complete peer network may cause the exogenous effects of the neighborhood physical environment to diminish. These are important questions for future research.

## Conclusions

Despite these limitations, our study presents some important considerations for both research on neighborhoods as well as for policies aimed at improving child behavior outcomes. With regards to research implications, our findings support the importance of carefully considering the geographic unit of analysis in studies of neighborhood effects on health. Although there have been limited attempts to examine the different geographic neighborhoods, most has relied upon existing administrative boundaries as the smallest geographic unit, be it census tracts in Canada [44,71], enumeration districts in the United Kingdom [72], or census blocks in the United States [73]. Only Chaix and colleagues in Sweden [43] examined the utility of a neighborhood definition that was completely independent from existing administrative boundaries. More research is needed to systematically compare neighborhood definitions and their relation to a variety of health outcomes. Recent advances in the use of such technology as Google Street View [74] may provide an opportunity for replicating this work in a range of urban and rural settings in different parts of the world.

Our findings also have implications for policies related to community-level interventions to reduce child behavior problems. In particular, failure to account for the three simultaneous paths of influence correctly may result in underestimation of the benefits of interventions. Our results indicate if the influence of neighborhood physical conditions and peer networks are accounted for, the potential economic impact of community-based parenting programs to prevent child behavior problems such as “Triple-P” [51] might be misestimated. A more comprehensive assessment of program impact is critical if policy makers are to compose informed decisions on how to improve population behavioral health in children and youth.

## Competing interest

The authors declare that they have no competing interests.

## Authors’ contributions

MC, TL, and JM were responsible for the conceptualization and design of the study, and TL was responsible for overseeing data collection. MC, TL, and KB were responsible for the analysis and interpretation of data and for drafting the manuscript. JM provided final approval of the manuscript. All authors read and approved the final manuscript.

## Supplementary Material

Additional file 1 Systematic social observational items at the parcel level. Click here for file
