# Supplementary material for: Defining neighborhood boundaries in studies of spatial dependence in child behavior problems
Source: Int J Health Geogr. 2013 May 3;12:24. doi: 10.1186/1476-072X-12-24 (PMC3648484; doi:10.1186/1476-072X-12-24)
Supplement: Additional file 1 — Systematic social observational items at the parcel level. [file 1476-072X-12-24-S1.doc]

Systematic social observational items at the parcel level

| **Item** | **Description** |
| --- | --- |
| Parcel usage | Single family, multi-family, commercial, parking lot, park, vacant, etc. |
| Compatibility of land use | Incompatible land use (e.g., residential next to junk yard or a vacant lot) |
| Area | Square feet |
| Condition of house | Good, cosmetic repairs, structural repairs, tear down condition |
| Peeling paint | Yes if present |
| Broken windows | Yes if present |
| Boarded windows | Yes if present |
| Barred windows | Yes if present |
| Barred doors | Yes if present |
| Uncovered crawl space | Yes if present |
| Condition of lawn | Well-kept or unkempt |
| Condition of fence | Well-kept or in poor shape |
| Trash on curb | Yes if bulk trash along the curb |
| Trash in yard | Yes if bulk trash or junk in the yard |
| Cars in yard/drive | Yes if vehicles in need of repair in the yard/drive |
| City citation (yard) | Yes if city code enforcement sign in the yard |
| City citation (house) | Yes if city code enforcement sign in window |
